# How do R&D and remittances affect economic growth? Evidence from middle-income countries

**DOI:** 10.1016/j.heliyon.2024.e30160

**Published:** 2024-04-22

**Authors:** Md Zahidul Islam, Sk Habibur Rahaman, Fuzhong Chen

**Affiliations:** a School of International Trade and Economics, University of International Business and Economics, Beijing, 100029, China; b Assistant Professor, DBA, Manarat International University, Dhaka, Bangladesh; c The Academy of China Open Economy Studies, University of International Business and Economics, Beijing, China; d School of International Trade and Economics, University of International Business and Economics, Beijing, China

**Keywords:** R&D, Remittances, Economic growth, Middle-income countries

## Abstract

Sustainable development through technical progress for middle-income countries (MICs) is overlooked in growth allied studies. Despite their crucial role in alleviating poverty and resource shortages, MICs encounter challenges in global economic competition, driving persistent efforts to find practical solutions. Therefore, this study explores the integrated impact of R&D expenditure and remittances on economic growth in MICs. Using data from 25 MICs between 1996 and 2021, this study employs the “2nd generation unit root” and “panel autoregressive distributed lag (ARDL)” methods. The “feasible generalized least square (FGLS)” techniques and the “Dumitrescu-Hurlin (D-H)” causality test are employed to verify the robustness of the panel ARDL estimation. The Westerlund cointegration tests confirm a long-term cointegration between variables. The findings of the panel ARDL approach show that R&D expenditure and remittances positively and significantly influence economic growth. The robustness of the panel ARDL results is confirmed by the FGLS estimation, which produces similar outcomes. The outcomes from the FGLS and the ARDL methods are additionally validated by the D-H causality check. Therefore, encouraging R&D and remittances is crucial to accelerate middle-income nations' economic growth. The study reveals a novel mechanism of R&D expenditures, remittances, and economic growth in MICs, shaping their mutual influence on this economic landscape. The study supports middle-income countries' policymakers in creating effective policies for their financial institutions regarding R&D expenditure and remittances.

## 1 Introduction

Economic growth differs among countries because of many global economic factors and cultural contexts. Distinguished world researchers have studied these issues over the last two decades. Still, most research has been done in OECD countries or developed countries due to the availability of reliable data on different determinants of economic growth. In order to achieve sustainable development, all nations must experience economic growth. Therefore, governments should consider which factors are essential for sustainable economic growth. Many countries encourage R&D and remittances to boost the economy and create advanced value-added performance. Still, there is little evidence for these issues in middle-income countries.

The selection of MICs and this topic is based on several reasons: First, MICs account for 75 % of the global population yet contribute just 33 % to global GDP [1]. Hence, the large population presents an opportunity to boost the economy by utilizing R&D sectors and creating skilled labor for other countries. Second, despite various methods and combinations of variables used in multiple studies, research on the relationship between R&D, remittances, and economic growth is limited. Third, most existing literature on these variables is primarily conducted in developed or OECD countries due to data availability, making it scarce in middle-income countries. Therefore, this study has the potential to significantly enhance the global economy by utilizing the large populations of MICs.

The main forces behind economic growth are labor, technical advancement, physical capital, and human capital, which economists understand well. In an economy, R&D spending and remittances are essential to economic growth. Endogenous growth models are utilized to analyze the correlation between GDP growth and R&D, focusing on technological advancement and innovative R&D components to prevent diminishing returns to scale. According to several scholars [[2], [3], [4], [5]], spending on R&D was a critical component in growing GDP. In contrast, some of them reached insignificant results [[6], [7], [8]]. Regrettably, research on the combined impact of R&D and remittances on economic growth in MICs is rare due to several challenges. Therefore, based on the above discussion, we hypothesize (H_1_) a strong and positive correlation between investing in R&D projects and the ensuing GDP growth in middle-income nations.

Previous studies provided an inclusive understanding of the correlation between remittances and economic growth, revealing positive and diverse associations. Some findings revealed a favorable association between remittances and economic growth [9,10], aligning with the endogenous growth theory. According to this theory, remittances can act as endogenous sources of income, fostering economic growth by stimulating consumption, investment, and overall economic activity within recipient countries. In contrast, the literature indicates significant and insignificant correlations between remittances and economic growth [[11], [12], [13]]. The relationship between remittances and economic growth is complex and influenced by factors like economic structure, policy environment, and efficient utilization of remittance funds. Using endogenous growth theory enhances our comprehension of remittances' role as intrinsic drivers of sustained economic growth. Based on the inclusive analysis above, we hypothesize (H_2_) that a positive relationship exists between remittances and economic growth in MICs. This proposition stems from the multifaceted discussion between these dynamic variables, suggesting a symbiotic relationship that contributes favorably to the economic paths of MICs."

On the other hand, just a few studies have demonstrated the modest cumulative impact of our chosen predictor variables on economic growth in various global contexts. For instance, First, Ref. [7], conducted a study in developing nations using several factors, including R&D, domestic investment, labor participation, human capital, FDI, and GDP. Second, Ref. [14], conducted comparative research in China to demonstrate the association between R&D, human capital, and FDI. Third, Ref. [15], investigated the connection between emerging countries' economic growth, remittances, foreign aid, and FDI. Fourth, related research demonstrates how remittances, government development aid, and FDI affect GDP in developing nations [12]. Contrary to that, our research focuses on the combined effects of R&D spending and remittances on GDP growth.

Integrating R&D expenditures with remittances offers a comprehensive method for measuring economic growth through various mechanisms. R&D expenditures drive innovation and technological progress, directly contributing to GDP enhancement by creating innovative products, processes, and knowledge spillover across industries. Simultaneously, remittances are financial inflows from overseas which are crucial in stimulating economic growth. Remittances are the source of funds for consumption, education, welfare, healthcare, noteworthy household income sources, and poverty alleviation, and they significantly affect the overall economic activity. In many countries, remittances are crucial for funding consumption, education, welfare, healthcare, household income, and overall economic activities. The combination of R&D and remittances provides a comprehensive view of innovation, socio-economic dynamics, improved living standards, and human capital development. By examining these variables jointly, this study seeks to clarify the interconnected mechanisms through which R&D expenditures and remittances contribute to a holistic economic growth approach.

The work advances earlier studies in several ways. First, we empirically investigate the affiliation between R&D expenditure, remittances, and GDP growth. We have already determined the separate investigations of R&D and remittances on GDP growth. However, few studies have demonstrated partially the individual and combined influence on economic growth. As a result, there is no evidence in the literature about the combined impact of R&D spending and remittances on GDP growth. Second, we determine the causal connection between R&D expenditure, remittances, and economic growth. This causal link between the chosen variables was primarily neglected in earlier investigations. Third, most earlier studies used a single or limited mix of our selected predictor variables partially with other variables to measure economic growth. Still, all that research was conducted in developed or OECD nations. Existing empirical studies indicate that there haven't been any investigations on this topic in middle-income countries. Therefore, this article has been undertaken to fill all the above gaps. Consequently, we hypothesize (H_3_) an optimistic and supportive effect of R&D spending, remittances, and GDP progress in the long run in MICs. We also hypothesize (H_4_) a bidirectional causative affiliation between the selected variables in MICs. Although the methodological approaches and context differ, we expect the findings to agree with the literature's theoretical and empirical results.

### 1.1 A synopsis of R&D expenditure, remittances, and economic growth in middle-income countries

Economic expansion should be a top priority for all nations. Despite the potential difficulties, achieving this goal may be challenging for middle-income countries. These nations frequently find themselves stuck in what is known as the “middle-income trap,” in which they cannot maintain high levels of economic growth and cannot keep pace with high-income nations. However, R&D expenditure is the latent factor for allocating financial resources to advance innovation, technology, and knowledge in MICs, demonstrating a commitment to progress and innovation. On the other hand, receipt of the remittances is a potential factor that can enhance the economy in MICs. They have the potential to not only serve as a source of revenue for households and companies but also as an investment vehicle for educational and medical facilities.

**Fig. 1:**
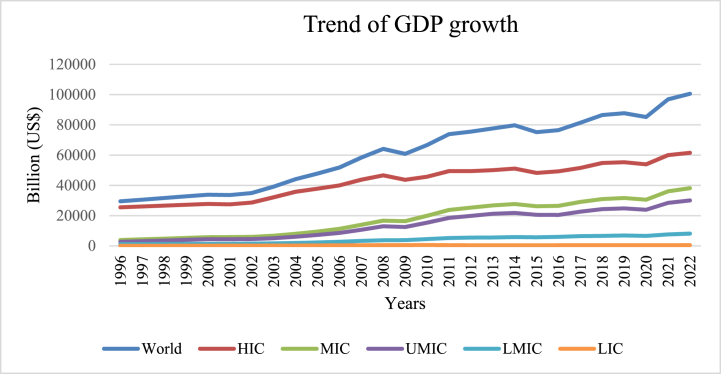
Trend of GDP growth.

The world's gross domestic product (GDP) expansion from 1996 to 2022 is depicted graphically in Fig. 1 [1]. Countries are labeled as either HIC (high-income countries), UMIC (upper-middle-income countries), MIC (middle-income countries), or LIC (low-income countries) on the graph. Global GDP has increased steadily over the past 27 years, with the highest growth occurring in the HICs. An estimated $120 trillion will be the world's GDP in 2023. There is a widening gap between HICs and the rest of the world, as the graph demonstrates.

**Fig. 2:**
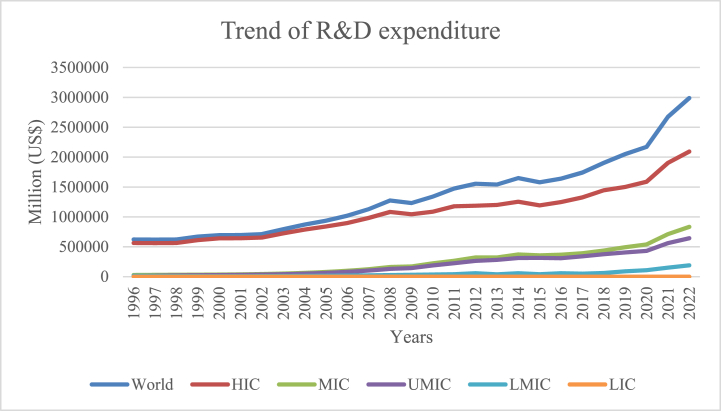
Trend R&D expenditures.

Fig. 2 displays the worldwide, middle, and low R&D spending trend from 1996 to 2022. The United States, Canada, Western Europe, Japan, and South Korea are the five high-income countries (HICs). HICs invest more heavily than MICs in research and development, giving them a head start in the scientific and technological fields.

**Fig. 3:**
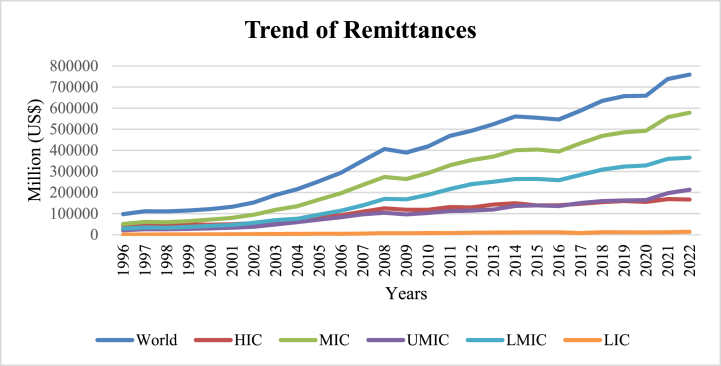
Trend of remittances.

Fig. 3 depicts the growth of remittance flows to different income groups of the world from 1996 to 2022 [1]. The figure also demonstrates the wide gap in remittance flows between HICs and the rest of the world. Compared to UMICs and MICs/LICs, HICs receive over three times as much in remittances inflows.

Considering the conversation above, hypotheses and data trends for the variables R&D, remittances, and economic growth in MICs determined that the effect of R&D and remittances on economic growth is indecisive and requires further research. Existing studies have offered insights into the individual impacts of R&D expenditure and remittances on economic growth. Still, there is a notable absence of comprehensive investigations into their combined effects within the exclusive context of MICs. Given the crucial role of these countries in the global economic landscape, understanding the synergies or divergences between R&D investment and remittance inflows becomes imperative. Therefore, this study seeks to address this gap by rigorously examining the concurrent influence of R&D expenditure and remittances on economic growth in MICs and identifying their mechanisms.

The current study provides significant contributions while attempting to fill some gaps. First, our research offers a novel theoretical contribution by integrating the impact of R&D spending and remittances on accelerating economic growth. Furthermore, this study provides unique evidence for a causal connection between GDP growth, R&D spending, and remittances. Second, this article reveals a contextual contribution as the latest experiential evidence for MICs due to the optimistic link between R&D outlay, remittances, and GDP progress. Third, the multivariate framework prevents missing data due to omitted variables. Fourth, the study explores the dynamic “R&D Expenditure-Remittances-GDP nexus” in MICs by incorporating control variables. The rest of the structure of this paper is as follows: literature review, methodology details with data set, analysis and empirical findings, discussion and implication, and conclusion with limitations.

## 2 Literature review

The review of relevant literature partially examines the relationship between R&D expenditure, remittances, and economic growth.

### 2.1 R&D expenditure and economic growth

Economic growth is a crucial topic for economists as it contributes to new economic thought and enhances production and capacity for many countries. The term “economic growth” is the primary prefix in Classical, Keynesian, Neoclassical, and Endogenous growth theories. Ref. [16], demonstrated that technical invention is a crucial element in long-term economic growth, which was ignored in classical and Keynesian economic theories. Ref. [17], posited that the accretion of production factors and advancement of technology were long-standing and control the diminishing return in Neo-classical growth theories. Concurrently, endogenous growth theories consider R&D spending for technological advancement, while neo-classical theories do not, highlighting the primary difference between these two approaches. Ref. [18], recognized and started work as part of endogenous theories that R&D expenditures are the fundamental element of economic progress. In endogenous growth models, R&D spending significantly influences economic growth. As a consequence, this study adheres to the principles of endogenous growth theory. This study utilizes the endogenous growth theory to comprehend the dynamic interplay of economic variables in the development process, focusing on the factors influencing sustained economic growth in MICs.

Since 1980, endogenous growth models have been utilized as theoretical tools to explore the rapport between R&D spending and GDP growth. Ref. [19] deemed the importance of high-tech advancement in R&D arrangements to be the key growth factor. According to the empirical literature based on macro and microeconomics around the globe, many studies were conducted with panel data over time in our selected independent variables individually. First, some researchers used time series or panel data, and they found an optimistic and statistically substantial effect of R&D expenditure on economic progress, including some control variables [[3], [4], [5],[20], [21], [22], [23], [24], [25], [26]]. Spending on R&D is essential for economic expansion, and the volume of GDP assigned to R&D positively impacts economic progress. For example, Ref. [5], discovered an optimistic connection between R&D, population growth, and GDP. The researcher directed R&D outlay as a ratio to GDP as increasing as economic expansion in OECD continues. Equally, Ref. [22], inspected “Research & development and growth by Bayesian model averaging analysis using data from 72 countries between 1960 and 1992.” The researcher realized that R&D spending supports economic expansion. Similarly, Ref. [23], used data from 1980 to 1998 with different variables such as kinds of R&D, government support, public institutions, and productivity growth in 16 OECD countries to demonstrate an optimistic and substantial influence of R&D allocation on economic advancement. Congruently, Ref. [25], established a link between R&D expenses and GDP expansion in emerging nations. The researchers discovered favorable and essential findings in all selected countries using data from thirty-two industrialized and twenty emerging nations from 1966 to 2010. Correspondingly, Ref. [26], utilized the GMM method to analyze data from seventeen OECD countries to determine the impact of R&D absorption in the health, chemical, medicine, electrical, and electronic sectors for innovation potential and production.

In contrast, adverse connections between economic growth and R&D spending were discovered by some researchers. For example, Ref. [6], examined the impact of technology on economic growth in 25 developing nations with the help of a random effect model with different tests and found a strong negative relationship between R&D spending and economic growth. Similarly, Ref. [7], examined the impact of R&D spending on the growth of developing economies between 2000 and 2009 using PMG, GMM, and 3sls-GMM models. Results showed negligible influence in LICs and favorable outcomes for UMICs. Correspondingly, Ref. [8], found insignificant results in a study that analyzed data from 2000 to 2006 in 30 developing countries. The study concluded that R&D expenses did not directly influence economic advancement in developing countries. However, the literature also revealed significant and insignificant outcomes in a study [20], who conducted multivariate analysis in 20 OECD countries and found a substantial correlation between R&D investment and GDP growth in G-7 nations but insignificant results in the overall sample.

### 2.2 Remittances and economic growth

This theoretical literature critically scans the nexus between remittances and economic growth through the lens of the endogenous growth theory [27]. Unlike traditional approaches, the endogenous growth theory explored the intrinsic nature of remittances as endogenous capital capable of fostering self-sustaining growth mechanisms. The endogenous growth theory provides a robust framework for understanding how remittances contribute to immediate consumption and investment and sustained development through cultivating human capital, technological improvement, and innovation [28]. This research synthesizes a theoretical foundation for analyzing the transformative potential of remittance inflows on long-term economic growth.

Previous research demonstrated a positive and substantial correlation between remittances and economic growth. For example, Ref. [9], examined the effect of remittances on GDP growth in six European countries with high remittances receipt rates between 1999 and 2013. The study found a strong and favorable link between remittances and growth in the selected nations. Similarly, Ref. [10], utilized the panel data (1977–2012) for four Asian countries to assess the association between GDP growth and migrant remittances. The study discovered a significant positive long-term correlation between remittances and economic growth in the designated countries. Ref. [27] consistently investigated how remittances affected GDP in 11 Caribbean countries between 1975 and 2011. They discovered the proof of diverse long-term causal linkages between the variables in seven nations.

In contrast, the existing literature acknowledged the mixed outcomes. For instance, based on the statistical analysis by Ref. [11], 538 assessments from 95 studies in middle- and low-income nations found that 40 % of studies indicated a significant impact of remittances on GDP growth, 20 % had a negative effect, and 40 % showed no effect. Similarly, Ref. [12] examined the impact of ODA, remittances, and FDI on economic growth in 41 emerging countries from 1990 to 2016. The study found that the financial flows ambiguous influenced economic growth. Congruently, Ref. [13] used a VAR framework and Granger causality test to observe the relationship between remittances and economic growth in Sri Lanka, India, and Bangladesh. Results demonstrated no direct link in India and a two-way causal connection in Sri Lanka.

### 2.3 R&D expenditure, remittances, and economic growth

The combination of R&D expenditure and remittances as measures for economic growth can be understood through the view of the endogenous growth theory. R&D expenditure drives technological innovation and productivity improvement, contributing to sustained economic growth by fostering knowledge, skills, and technology advancements. On the contrary, remittances, representing funds transferred by expatriates to their home countries, inject stable and often countercyclical financial resources into the economy. The synergy rises when R&D activities are fueled by domestic investments and the supplementary financial constancy provided by remittances. Remittances act as a buffer against economic surprises, supporting the continuity of R&D initiatives even in challenging periods. The combination of innovation and financial resilience in this dual approach has the potential to foster robust and sustained economic growth. The endogenous growth theory further emphasizes that innovation and technological progress can be self-reinforcing, generating a positive response loop and contributing to long-term economic development.

The literature highlights studies that smartly combine R&D expenditures with other variables for measuring economic growth. Such as the first, Ref. [14], examined the connections between economic growth, R&D, FDI, and human capital in China between 1991 and 2015. The findings indicated that R&D and FDI connected to human capital have short- and long-term effects on GDP. Second, Ref. [29], discovered the impact of government efficacy and R&D spending on economic growth in four African nations from 2000 to 2016, revealing that governance and R&D spending were crucial for economic growth. Third, Ref. [30], identified the influence of R&D, FDI, and intellectual property rights on economic growth in 92 nations from 1970 to 2007. Results showed that FDI, human capital, trade openness, R&D capability, local investment, and IPR protection positively impact economic growth.

On the other hand, literature integrates remittances with diverse factors for determining economic growth. For example, depending on the endogenous growth theory, Ref. [15] studied 53 African and 34 USA and Caribbean nations to see how GDP was affected by remittances, FDI, and foreign aid. The study revealed that geographical interconnectedness significantly influenced economic growth, as one economy relied on its neighbors. Second, Ref. [31], explored the effect of crude oil prices, personal remittances, and foreign debt on India's economic development using the DARDL simulations framework. Results showed that remittances hindered long-term growth, oil prices positively impacted growth, and foreign debt decreased growth. Third, Ref. [32] examined Ghana's economic development from 1980 to 2018 using the panel ARDL method. The study found that remittances positively impacted growth in both the short and long term, while imports, real exchange rates, and FDI had an adverse effect on the economy.

Based on the above discussion from the existing literature, it is found that research on the combined impact of R&D and remittances on economic growth is notably rare. To the best of our knowledge, the current literature on the intersection of these two variables is infrequent and lacks empirical evidence in the context of MICs. Therefore, this study aims to investigate the impact of R&D and remittances on economic growth in MICs.

## 3 Materials and methods

### 3.1 Source of data and information about the variables

This article is considered a panel of 25 middle-income countries (MICs)* from 1996 to 2021. The countries from MICs have been chosen based on the available data from the selected sources. Data from other MICs for the particular independent variables are unavailable in the sources. Therefore, countries from the middle-income range are chosen using the convenience sample approach. GDP is the dependent variable, R&D expenditure and remittances are the independent variables, and FDI, capital stock, total factor of productivity, and labor force participation are the control variables for this study. The logarithm form maintains smoothness, prevents outliers, reduces autocorrelation and heteroskedasticity effects, and prevents erroneous regressions for all variables. Table 1 presents details regarding the definition and source of variables.

**Table 1:** Definition of variables.

Variable	Description	Source
LnGDP	Log value of GDP per capita in current US dollars	WDI
LnRD	Log value of R&D expenditures in current US dollars, (after calculating the given percentage from GDP)	
LnFR	Log value of foreign remittances (personal) received in current US$	
LnFDI	Log value of foreign direct investment (inflow) in current US$	
LnLBO	Log value of Labor force participation as % of total population ages 15+	
LnTFP	Log value of total factor of productivity as the constant national price (TFP level at current PPPs-USA = 1)	PWT 10.01
LnKS	Log value of the capital stock at constant 2017 national prices (in 2017 US$)	

Source [1,33]: Authors' compilations. *Note: Upper-MIC - Malaysia, Kazakhstan, Turkey, Armenia, Serbia, Peru, Thailand, Colombia, Costa Rica, Mexico, Paraguay, Bulgaria, Russian Federation, China, South Africa, Brazil, and Argentina; Lower-MIC - Tunisia, Mongolia, Kyrgyz Republic, Tajikistan, India, Egypt, Ukraine, Iran [1].

### 3.2 Model specification

Endogenous growth theory and experiential studies in the previous works have shaped the study's central tenet: “There is a significant correlation between R&D expenditure, remittances, and economic growth.” This hypothesis is expressed in a purposeful equation (1) below:

(1) GDP=∫(RD,FR,Z)

This functional connection between the explained and explanatory variables has been tested with an econometric model using panel data of middle-income countries in a logarithmic system for efficient estimation in equation (2).

(2) $${LnGDP}_{it}=\beta_{0}+\beta_{1}{LnRD}_{it}+\beta_{2}L{nFR}_{it}+{\vartheta Z}_{it}+\mu_{it}+\varepsilon_{it}$$

Where ε means the residual term, t means time, and i mean countries. LnGDPit (Log Gross Domestic Product) is the primary dependent variable. LnRDit and LnFRit are the independent variables. Zit is the matrix of control variables, including LnFDIit, LnTFPit, LnKSit, and LnLBOit. ***μ***it and εit are the unknown country/time-specific effects and the error term, respectively.

The CD test identifies cross-sectional dependence (CD) in the panel dataset. This study uses different contemporary tests consecutively, such as “Breusch and Pagan (BP) [34] LM test, Pesaran [35] Scaled LM test, and Pesaran [35] CD test” for finding out the CD. Considering the unreliable results from the conventional test, we employ “second-generation unit-roots tests, such as CIPS by Ref. [36] and CADF by Ref. [37]. After validating the cointegration linkages among the variables, we apply panel cointegration tests of [38] to check the long-run cointegration in the variables. The slope heterogeneity and panel dependency are permitted in this test for the panel variables.

However, after confirming the expected properties of the panel data, we use the “panel autoregressive distributed lag (ARDL) method” to measure the affiliation between R&D spending, remittances, and GDP growth in MICs. The panel ARDL technique can be evaluated using panel error correction (ECM) models [39], considering country-specific heterogeneity apprehension in the short and long term. For several reasons, the panel ARDL model is favored over the single cointegration technique for determining a long and short-term equilibrium. Furthermore, the most reliable long-run and short-run coefficients are provided by the panel ARDL model [[40], [41], [42]]. Given these merits, this article uses the panel ARDL approach to examine the long and short-term association among R&D expenditure, remittances, and economic growth.

Both AIC and SBC determine one lag length for the series. Consequently, the panel ARDL technique created by Pesaran et al. in Ref. [39] is in equation (3):

(3) $$Y_{it}=\alpha +\sum_{j=1}^{p-1}\gamma_{y}^{i}{\left(y_{i}\right)}_{t-j}+\sum_{j=0}^{q-1}\delta_{y}^{i}{\left(x_{i}\right)}_{t-j}+\mu_{i}+\varepsilon_{it}$$

Where $Y_{it}$ is the regress (LnGDP), $X_{it}$ are regressors (LnRD, LnFR, etc.) where k × 1 vectors are permitted to be decently I (0) or I (1) or co-integrated. “The coefficient of the lag-dependent variable is $\gamma_{it}$; then $\delta_{it}$ are k × 1 coefficient vectors; the unit-specific fixed effects are denoted by $\mu_{i}$; and *i* = 1 …, *N; t* = 1, 2, …, *T; p, q* are optimal lag orders; finally, $\varepsilon_{it}$ is the error term.”

The following diagram (equation (4)) represents the re-parametrizes ARDL error correcting model:

(4) $${\Delta Y}_{it}=\alpha_{0}+\sum_{j=1}^{p-1}\gamma_{y}^{i}\Delta {\left(Y_{i}\right)}_{t-j}+\sum_{j=0}^{q-1}\delta_{y}^{i}\Delta {\left(x_{i}\right)}_{t-j}+\theta_{i}\left[{Y_{i,}}_{t-1}-\beta_{i}^{\prime }{X_{i}}_{,t-1}\right]+\mu_{i}+\varepsilon_{it}$$

Where Δ = The first differences operator

$\theta_{i}=-\left(1-\gamma_{i}\right)$, group-specific (error correction) speed of adjustment coefficient.

$\beta_{i}^{\prime }$ = Vector of long-run relationships

$ECT=\left[{Y_{i,}}_{t-1}-\beta_{i}^{\prime }{X_{i}}_{,t-1}\right]$, the error correction term

$\gamma_{y}^{i},\delta_{y}^{i}$ = are the short-run dynamic coefficients

For estimating the short- and long-term impact of R&D expenditure and remittances on economic growth, this study uses panel error correction models (ECM). According to ECM's instruments, the selected explained and explanatory variables show long- and short-term relationships. Equation (4) can be examined using the panel ARDL estimator because the maximum likelihood is utilized to explore “the long-run equilibrium and heterogeneity of the dynamic adjustment process” [43].

The panel ARDL model's reliability is verified using several diagnostic techniques. The results of the panel ARDL approach is further examined using the FGLS approach. The robustness analysis uses the efficient and homoscedastic FGLS method, which effectively handles heteroskedasticity and serial correlation issues. If the result is heteroscedastic, we divide both sides of equation (2) by $\delta_{i}$ and rewrite equation (5).

(5) $$\frac{{LnGPD}_{it}}{\delta_{i}}=\frac{\beta_{0}}{\delta_{i}}+\frac{\beta_{1}{LnRD}_{it}}{\delta_{i}}+\frac{\beta_{2}{LnFR}_{it}}{\delta_{i}}+\frac{\vartheta Z_{it}}{\delta_{i}}+\frac{\mu_{it}}{\delta_{i}}+\frac{\varepsilon_{it}}{\delta_{i}}$$

Equation (5), which we simplify and employ in this work as the main reference equation for the econometric analysis, replaces equation (6) in a homoscedastic and effective approach.

(6) $${LnGDP}_{it}^{*}=\beta_{it}^{*}+\beta_{1}{LnRD}_{it}^{*}+\beta_{2}{LnFR}_{it}^{*}+\vartheta Z_{it}^{*}+\mu_{it}^{*}+\varepsilon_{it}$$

The panel estimators presented above and explained above do not give enough evidence for a causal link in a panel data set. The panel causality analysis of “Dumitrescu-Hurlin (DH)” has been utilized to clarify the causal connection between the selected variables. The test can analyze imbalanced panel data while considering the time dimension and cross-section size. “It performs effectively when slope heterogeneity and cross-sectional dependence between countries are present [44,45].” A brief overview of the D-H model is presented below in equation (7).

(7) $$Y_{it}=\beta_{i}+\sum_{i=1}^{k}\alpha_{i}Y_{i,t-k}+\sum_{i=1}^{k}\delta_{i}X_{i,t-k}+\varepsilon_{it}$$

In this case, $\alpha_{i}$ stands for the lag parameter, $\delta_{i}$ for the coefficient slope and $\beta_{i}$ stands for the constant. The null and alternative hypotheses are both defined with clarity in equation (8).

(8) $$H_{0}:\delta_{i}=\delta_{i}=0,H_{i}:\left\{ \begin{array}{c} \delta_{i}=0,\forall_{i}=1,2,\ldots \ldots \ldots \ldots \ldots \ldots \ldots \ldots \ldots \ldots N\\ \delta_{i}\neq 0,\forall_{i}=N_{1}+1,{\;N}_{1}+2,\ldots \ldots \ldots \ldots .N \end{array}\right.$$

Although all cross-sections show non-homogenous Granger causality, the alternative hypothesis contends that panel data may be used to identify at least one causal relationship.

Furthermore, due to its ability to accommodate a variety of lag configurations that include both the stationary and dynamic results of the selected factors, the panel ARDL methodology has recently grown to be a very effective and popular econometric estimating technique [46,47]. Correspondingly, the D-H model with “the panel ARDL and the FGLS techniques” provide valid estimates for long- and short-run causal associations, enhancing researchers' understanding of component interactions [48].

## 4 Results and discussion

### 4.1 Panel unit-root and CD test results

Table 2 provides a summary of the unit root test results. The study uses CIPS and CADF from second-generation tests attributable to the presence of CD to find out the data stationarity of all the selected variables (LnGDP, LnRD, LnFR, LnFDI, LnTFP, LnKS, and LnLBO). At the 1 % level of significance, all variables are stationary at various integrating orders or a mix of I (1) and I (0), but no one I (2) factors in the variables. In this case, the panel ARDL model is suitable for producing reliable results.

**Table 2:** Panel unit root test.

	CADF		CIPS	
Variables	At level	First difference	At level	First difference
LnGDP	−2.739^a^	−3.341^a^	−2.545^a^	−4.211^a^
LnRD	−2.475^a^	−3.784^a^	−2.376^a^	−4.276^a^
LnFR	−2.576^a^	−3.212^a^	−2.210^b^	−4.485^a^
LnFDI	−2.365^a^	−4.207^a^	−2.925^a^	−5.454^a^
LnTFP	−1.531	−2.911^a^	−1.512	−4.273^a^
LnKS	−2.252^a^	−2.190^b^	−1.303	−3.056^a^
LnLBO	−1.947	−3.042***	−1.604	−3.632^a^

a p < 0.01.

b p < 0.05.

Before doing the panel ARDL estimation, “cross-sectional dependency (CD) tests, panel unit root tests, and cointegration tests have been run on the panel data to look for serial correlation, cross-sectional dependence, and unit root [49].” Due to the similar nature of economic development of selected countries, the CD tests are essential. The article uses “Pesaran CD [35], Pesaran scaled LM and Breusch-pagan LM tests” for CD. Table 3 presents the empirical outcomes for CD. Due to the findings, “the null hypothesis is rejected at a 1 % significance level for each of the separate tests in the series.” That means CD exists in the selected variables due to interconnected elements, including globalization, macroeconomic prospects, and economic targets of the nations.

**Table 3:** Results of CD test.

Test	BP-LM	Pesaran S-LM	Bias cor. S-LM	Pesaran CD
d.f	300			
LnGDP	6631.01^a^	258.46^a^	257.97^a^	81.22^a^
LnRD	5459.75^a^	210.64^a^	210.14^a^	72.83***
LnFR	5190.66^a^	199.66^a^	199.16^a^	61.27^a^
LnFDI	2663.44^a^	96.48^a^	95.98^a^	46.49^a^
LnTFP	3019.83^a^	111.03^a^	110.53^a^	17.49^a^
LnKS	5797.27^a^	224.42^a^	223.92^a^	73.29^a^
LnLBO	1720.90^a^	58.00^a^	57.50^a^	2.85
Residual-based CD	1552.90^a^	51.14^a^	–	6.61^a^

a p < 0.01.

### 4.2 Descriptive statistics

Table 4 represents the descriptive statistics, including all selected factors (LnGDP, LnRD, LnFR, LnFDI, LnTFP, LnKS, and LnLBO) from 1996 to 2021. Six hundred fifty observations make up the sampled data (N*T), including central tendency and measures of dispersion such as mean, median, SD, etc. The median and mean variables are nearly in perfect agreement, indicating minimal inconsistency and symmetry. LnGDP has an average value of 11.03, and LnRD is 8.63. Moreover, the maximum and lowest values of each variable are also shown by the Max and Min values. Standard deviation (SD) is determined by “the sum of squared deviations from the mean,” such as the SD of LnFDI is 0.89. The statistics show that the study necessitates that all chosen components have positive means and impressively stable distributions. The analysis requires that all selected variables have uniform distributions and positive means.

**Table 4:** Summary statistics.

Vars	Obs	Mean	Median	Max	Min	SD
LnGDP	650	11.038	11.131	13.301	8.935	0.882
LnRD	650	8.632	8.672	11.661	5.489	1.153
LnFR	650	9.152	9.206	11.070	6.042	0.803
LnFDI	650	9.449	9.520	11.826	6.669	0.893
LnTFP	650	−0.274	−0.244	0.129	−0.786	0.178
LnKS	650	11.944	12.044	14.083	9.216	0.913
LnLBO	650	1.766	1.776	1.902	1.602	0.069

### 4.3 Correlation matrix and multicollinearity test

Table 5 depicts the correction matrix, including all selected variables for this article. It indicates a significant positive association among R&D spending, remittances, FDI, productivity, capital stock, labor, and economic growth. Furthermore, no multicollinearity is associated among variables for measuring economic growth.

**Table 5:** Correlation matrix.

Var	LnGDP	LnRD	LnFR	LnFDI	LnTFP	LnKS	LnLBO
LnGDP	1.00						
LnRD	0.96^a^	1.00					
LnFR	0.59^a^	0.56^a^	1.00				
LnFDI	0.88^a^	0.86^a^	0.57^a^	1.00			
LnTFP	0.36^a^	0.35^a^	0.21^a^	0.24^a^	1.00		
LnKS	0.65^a^	0.62^a^	0.45^a^	0.52^a^	0.07^b^	1.00	
LnLBO	0.18^a^	0.11^b^	−0.13^a^	0.26^a^	−0.25^a^	0.03	1.00

a p < 0.01.

b p < 0.1.

Table 6 shows the variance inflation factor (VIF) outcomes based on baseline equation (2). We discover the mean VIF of 2.80, and the value of an individual factor is below 5.5 in the case of all variables. Therefore, no multicollinearity issue in our model.

**Table 6:** Variance inflation factor (VIF).

Variable	VIF	1/VIF
LnRD	5.45	0.18
LnFDI	5.02	0.20
LnFR	1.77	0.56
LnKS	1.77	0.56
LnLBO	1.41	0.70
LnTFP	1.34	0.74
Mean VIF	2.80	

### 4.4 Panel cointegration test results

After establishing the long-term relationship between the variables using unit root tests, we further examine the connection using the Westerlund cointegration tests.

Table 7 shows the result of Westerlund's (2007) cointegration test where the Gt, Pt, and Ga, Pa tests for the panel of MICs reject the null hypothesis that there is no cointegration at 1 % based on robust p-value by bootstrap. Therefore, cointegration exists in the panel, meaning long-term relationships exist in the selected variables.

**Table 7:** Westerlund cointegration test results.

	Value	Z-value	P-value	Robust P-value
Gt	−2.424^a^	2.143	0.984	0.000
Ga	−7.026^a^	5.645	1.000	0.000
Pt	−12.482^a^	0.372	0.645	0.000
Pa	−6.955^a^	3.534	1.000	0.000

a p < 0.01.

### 4.5 Panel ARDL estimation results

The PMG-ARDL model is widely utilized in various economic applications. According to the Akaike Information Criterion, a model with a maximum of one lag for dependent and two lags for independent variables is ARDL (1, 2, 2, 2, 2, 2, 2). Table 8 shows the PMG-ARDL model's output.

**Table 8:** Panel ARDL's long and short-term results.

Variable	Coef	S. E	t-Stat	P-value
Long Run Equation				
LnRD	0.210^a^	0.027	7.662	0.000
LnFR	0.171^a^	0.022	7.870	0.000
LnFDI	0.062^a^	0.020	3.048	0.003
LnTFP	1.467^a^	0.150	9.766	0.000
LnKS	0.624^a^	0.043	14.557	0.000
LnLBO	3.032^a^	0.441	6.945	0.000
Short Run Equation				
COINTEQ01	−0.213^a^	0.060	−3.544	0.001
D (LnRD)	0.241^a^	0.041	5.910	0.000
D (LnFR)	−0.004	0.064	−0.064	0.949
D (LnFDI)	0.030^b^	0.014	2.161	0.032
D (LnTFP)	0.192	0.269	0.715	0.475
D (LnKS)	0.571^c^	0.319	1.793	0.074
D (LnLBO)	−0.178	0.607	−0.293	0.770
C	−1.131^a^	0.326	−3.472	0.001

a p < 0.01.

b p < 0.05.

c p < 0.1.

The study demonstrates that all nominated regressors positively affect regress in the long run. According to PMG outcomes, a 1 % increase in R&D expenditure corresponds to a long-term economic growth augmentation of 0.21 %. Therefore, the study exposes a beneficial association between R&D and economic growth in MICs. The study partially aligns with previous research by other researchers [5,25], who explored similar relationships. Within the spectrum of researchers, Ref. [5], specifically focused on OECD countries by incorporating other independent variables with R&D, providing a comprehensive analysis that extends beyond a singular focus. Similarly, Ref. [25], found that R&D spending has a beneficial influence on economic development in all developed countries over the long run but a poor effect in the short run for developing nations. R&D spending is linked to higher economic growth, indicating that countries that invest more in it tend to perform better, leading to the development of new technologies and products that can boost economic progress. However, the literature also revealed differing results from Refs. [6,8], who found that R&D expenditure hinders economic growth.

The coefficient of remittances (LnFR) demonstrates a significant and positive long-term impact on GDP in MICs. The outcome aligns with results from Refs. [9,10], and disparate partially from the findings of [11,12] due to variations in remittance levels and the inclusion of different income group countries, respectively, albeit with differing magnitudes. Studies [9,10], indicated a strong link between remittances and economic development in developed countries, highlighting the significant role of remittances in driving comprehensive economic growth; contradictorily, other studies [11,12] identified both negative and positive associations, encompassing diverse income groups of countries.

The study reveals a significant positive correlation between FDI and economic growth in MICs. It supports previous research by Ref. [50], suggesting that FDI inflow contributes significantly to inclusive economic growth in the long term. Like FDI, productivity (TFP) demonstrates the positive impact on GDP at 1 % significance. These results significantly support stimulating economic growth in MICs. According to the finding, an average 1 % rise in LnTFP resulted in an additional 1.46 % average yearly advances in economic growth in the long run, *ceteris paribus*. The empirical finding of productivity aligns with key macroeconomic theories and literature, partially supported by Refs. [51,52] respectively. Consequently, our research demonstrates a distinct and substantial affiliation between capital stock (LnKS) and GDP, which is partially related to the conclusion of Ref. [53].

Subsequently, labor force participation (LnLBO) in economic growth varies over time. Labor force participation has a mixed effect on GDP, depending on various development-related parameters, including income level and gender. The connection between labor force participation and GDP is U-shaped [54,55]. Literature also revealed both positive [56] and negative [57] relationships between economic growth and labor force participation. In this study, the coefficient of labor force participation in MICs with growing economic growth is claimed to have a positive impact based on the context and cultural differences in this region. The findings ratify that all selected variables positively impact GDP progress in middle-income countries over the long term. Therefore, the study is crucial for promoting economic growth for MICs where R&D expenditure and remittances are vital.

In addition, the study reveals that R&D, FDI, and capital stock (LnKS) are the most significant contributors to economic growth in the short run. In contrast, the influence of other variables is shown to be negligible. Long-run associations between the endogenous and exogenous variables are discovered by the mark of “Error correction term (ECT) coefficient, which is [cointeq (−1)].” According to the ECT coefficient of −0.210, the rate at which economic growth levels deviate from the short and long-run equilibrium is about 0.21 percent yearly. As a result, the panel shows the long-run connotation between LnGDP, LnRD, LnFR, LnFDI, LnTFP, LnKS, and LnLBO. However, the long-run influence of all selected independent variables on GDP progress is more significant than the short-run impact.

### 4.6 Robustness analysis

The feasible generalized least squares (FGLS) regression is a statistical method used to estimate and authenticate the long-term results of the panel ARDL technique. Table 9 shows the outcomes of the FGLS approach, where we apply four models (Model 1 to Model 4) to determine the impact of R&D and remittances on economic growth in MICs. Models 1 and 2 represent the results for all variables, including year dummy and country dummy, respectively. Model 3 represents the findings for all factors, including both dummy variables, while model 4 represents the outcomes without any dummy variable. However, the study consistently reveals positive and significant results for all explained variables in all four models, including and excluding dummy variables. Therefore, by running four different models with different combinations of dummy variables, the outcomes obtained in each model are significantly robust. Consequently, we obtain a consistent and parallel result from the method with panel ARDL estimation based on theory and the coefficient's sign.

**Table 9:** FGLS outcome for robustness.

VAR	Model 1	Model 2	Model 3	Model 4
LnRD	0.565^a^	0.317^a^	0.256^a^	0.569^a^
	(0.0142)	(0.0180)	(0.0173)	(0.0140)
LnFR	0.0935^a^	0.0853^a^	0.0678^a^	0.0898^a^
	(0.0121)	(0.00838)	(0.00837)	(0.0114)
LnFDI	0.0965^a^	0.0704^a^	0.0616^a^	0.0908^a^
	(0.0179)	(0.00869)	(0.00818)	(0.0173)
LnTFP	0.391^a^	0.793^a^	0.620^a^	0.395^a^
	(0.0450)	(0.0497)	(0.0470)	(0.0447)
LnKS	0.0889^a^	0.435^a^	0.257^a^	0.0901^a^
	(0.00994)	(0.0277)	(0.0313)	(0.0100)
LnLBO	1.378^a^	0.0808	−0.293	1.395^a^
	(0.117)	(0.198)	(0.179)	(0.118)
Year Dummy	Yes		Yes	
Country Dummy		Yes	Yes	
Constant	1.072***	1.763***	5.336***	1.018***
	(0.228)	(0.447)	(0.525)	(0.224)
Observations	650	650	650	650
Number of countries	25	25	25	25

a p < 0.01.

The research continues by examining the causal relationship between the chosen explanatory and control variables. Only the direction and result of the relationship are provided by the long-term coefficients calculated by applying the panel ARDL and the FGLS approaches. However, they are useless for establishing any sort of causal connection. Table 10 shows the causal relationship between the selected variables.

**Table 10:** Pairwise D-H panel causality tests outcomes.

SL	H_0_	W-Stat.	Zbar-Stat.	p-value	Results
**1**	LnRD → LnGDP	2.105	2.990	0.003	LnRD ↔ LnGDP
	LnGDP → LnRD	2.735	4.866	0.000	
**2**	LnFR → LnGDP	7.086	17.814	0.000	LnFR ↔ LnGDP
	LnGDP → LnFR	2.624	4.536	0.000	
**3**	LnFDI → LnGDP	2.955	5.520	0.000	LnFDI ↔ LnGDP
	LnGDP → LnFDI	3.990	8.600	0.000	
**4**	LnTFP → LnGDP	3.180	6.189	0.000	LnTFP ↔ LnGDP
	LGDP → LTFP	7.020	17.615	0.000	
**5**	LnKS → LnGDP	3.601	7.441	0.000	LnKS ↔ LnGDP
	LnGDP → LnKS	7.560	19.223	0.000	
**6**	LnLBO → LnGDP	4.830	11.099	0.000	LnLBO ↔ LnGDP
	LnGDP → LnLBO	6.178	15.111	0.000	

Note: Bidirectional relationship (↔).

### 4.7 D-H panel causality results

The findings of the “D-H″ panel's pairwise causality test on heterogonous panels are displayed in Table 10, which yields twenty bidirectional and one unidirectional causal link. To test the reliability of the panel ARDL estimates, we still provide just the top six causalities linked with GDP. However, the one unidirectional causation is LnRD→LnTFP.

According to the findings, all the selected variables have bidirectional causation with GDP, meaning that R&D outlay, remittances, FDI, productivity, capital stock, and labor positively impact economic growth and vice versa. On the other hand, the causal link between all other selected variables is also bidirectional except LnRD and LnTFP, which means they positively affect one another. The study reveals only one unidirectional relationship (LnRD→LnTFP), which implies R&D causes total factor of productivity. The outcomes of the panel ARDL and the FGLS methods are verified as accurate, and their robustness is demonstrated by the twenty feedback and one unidirectional causation. Consequently, the projected results are reliable and suitable. Therefore, the series proves the presence of causation among the selected explanatory and explained variables. So, the study concludes that increased R&D and remittances in middle-income countries influence economic growth.

## 5 Conclusion and policy implications

The study investigates the effect of R&D expenditure and remittances on GDP expansion in MICs from 1996 to 2021, highlighting the potential for increased economic growth. The research applies second-generation unit root tests for finding data stationarity and the second-generation Westerlund cointegration methods for identifying cointegration among the variables. The study applies the panel ARDL and the FGLS econometrics approaches to examine the relationship between R&D, remittances, and GDP progress in MICs. The FGLS method is used to check the robustness of the results. “The heterogeneous panel D–H Granger causality test” is also executed in this research to determine the causal relationship among the chosen factors.

The empirical findings from the panel ARDL model highlight the long-run positive link between R&D, remittances, and GDP enrichment in MICs. The outcomes obtained through the FGLS method corroborate the findings of the panel ARDL approach, demonstrating consistency and reliability. The paired D-H panel causality tests also confirm the research outcomes of the ARDL and the FGLS methods. The causality tests reveal a bidirectional causative link between R&D spending, remittances, and economic growth. The findings of the causative analysis show that R&D expenditure and remittances enhance GDP in MICs. As a result, the empirical findings and discussion of the individual country support the idea that R&D expenditure and remittances are all positively correlated in middle-income countries.

Because of empirical discoveries, the subsequent policy actions are suggested: The governments of middle-income countries ought to strategically formulate policies that allocate ample funds to R&D, fostering technological and infrastructural advancements, thereby propelling sustained long-term economic growth. Governments also should encourage remittances to boost R&D investments, creating a mutually reinforcing cycle. This ensures stable external inflows and continuous investments in cutting-edge developments, fostering a more robust and dynamic economic landscape for MICs. The stability and additional financial resources from remittances contribute to advancements in technology and innovation spurred by R&D. This strategic approach ensures a more robust and dynamic economic landscape for MICs. Corporate managers strategically promote organizational growth by regularly acquiring R&D sectors through remittances. This strategic approach ensures continuous financial resources are injected into R&D activities, enabling companies to stay ahead of innovation and technological advancements. Integrating remittances into R&D investments provides external funding stability and fosters long-term growth, allowing the companies to navigate dynamic market landscapes, remain competitive, and contribute to the economic development of middle-income countries. The evidence suggests that investors can benefit from a dual strategy of R&D investments and remittances, which fosters innovation and technological advancements and ensures a stable financial foundation. This approach allows investors to participate in economic growth with a nuanced approach, capitalizing on the synergies between innovation-driven development and remittance inflows. This dual focus enhances the potential for a robust and sustainable return on investment, making it an attractive proposition for wealth creation. Overall, middle-income countries can have the possibility to utilize their vast human resources potential by implementing the above recommendations that promote R&D investments, encourage remittances, and cultivate innovation. This strategic approach unlocks untapped human capital, fostering the establishment of novel sectors that can significantly contribute to economic growth and development in these countries. Researchers can capitalize on the synergistic impact of R&D and remittances on economic growth as pioneering evidence, enriching existing knowledge and presenting new proof across diverse settings with varied approaches. This strategic utilization enhances the robustness and applicability of findings, contributing to a subtle understanding of the interconnected dynamics between these variables in fostering economic growth.

The novel contributions of this study to economic literature are as follows. First, this fundamental study offers a unique contribution to the existing literature by examining the combined effects of R&D and remittances on economic growth, which is rare in previous studies. Second, this study marks a pioneering contextual contribution to economic literature by exploring the combined impacts of R&D and remittances on GDP growth in middle-income countries. Third, the study's findings offer policymakers in MICs an opportunity to stimulate economic growth by endorsing R&D spending and promoting remittances through appropriate policies.

Although the findings are surprising and may have far-reaching policy implications, this study isn't without its flaws. Only 25 of the world's middle-income nations are included in the research. The variables and cross-sections may be expanded in the future to study the symmetric and asymmetric impact of R&D, remittances, FDI, and productivity on economic expansion. Other potential independent variables in this line of inquiry may include energy sources, information and communication technologies, and market liberalization.

## Funding statement

This research was funded by the General Research Fund of Beijing Social Science Foundation (Grant No. 23JJB009).

## Data availability statement

Data related to this investigation have been posted at https://databank.worldbank.org/source/world-development-indicators, an open-source online data repository hosted at The World Bank.

https://www.rug.nl/ggdc/productivity/pwt/?lang=en, an open-source online data repository hosted at The Groningen Growth and Development Centre (GGDC).

## CRediT authorship contribution statement

**Md Zahidul Islam:** Investigation, Formal analysis. **Sk Habibur Rahaman:** Data curation, Conceptualization. **Fuzhong Chen:** Supervision.

## Declaration of competing interest

The authors declare that they have no known competing financial interests or personal relationships that could have appeared to influence the work reported in this paper.
